# The use and impact of digital COVID-19 tracking in adult social care: a prospective cohort study of care homes in Greater Manchester

**DOI:** 10.1186/s12879-022-07939-6

**Published:** 2023-01-23

**Authors:** Akbar Ullah, William Whittaker, Fay Bradley, Pauline A. Nelson, Dawn Dowding, Marcello Morciano, Nicky Cullum

**Affiliations:** 1 Manchester Centre for Health Economics, Faculty of Biology Medicine and Health, The University of Manchester, Jean McFarlane Building, Oxford Road, Manchester, M13 9PL UK; 2 Division of Nursing, Midwifery and Social Work, School of Health Sciences, Faculty of Biology Medicine and Health, The University of Manchester, Jean McFarlane Building, Oxford Road, Manchester, M13 9PL UK; 3 Department of Economics, University of Modena and Reggio Emilia, Via Università, 4, 41121 Modena, MO Italy

**Keywords:** Care homes, Digital tracker use, COVID-19, Impact evaluation, Panel data, Greater Manchester

## Abstract

**Background:**

To support proactive care during the coronavirus pandemic, a digital COVID-19 symptom tracker was deployed in Greater Manchester (UK) care homes. This study aimed to understand what factors were associated with the post-uptake use of the tracker and whether the tracker had any effects in controlling the spread of COVID-19.

**Methods:**

Daily data on COVID-19, tracker uptake and use, and other key indicators such as staffing levels, the number of staff self-isolating, availability of personal protective equipment, bed occupancy levels, and any problems in accepting new residents were analysed for 547 care homes across Greater Manchester for the period April 2020 to April 2021. Differences in tracker use across local authorities, types of care homes, and over time were assessed using correlated effects logistic regressions. Differences in numbers of COVID-19 cases in homes adopting versus not adopting the tracker were compared via event design difference-in-difference estimations.

**Results:**

Homes adopting the tracker used it on 44% of days post-adoption. Use decreased by 88% after one year of uptake (odds ratio 0.12; 95% confidence interval 0.06–0.28). Use was highest in the locality initiating the project (odds ratio 31.73; 95% CI 3.76–268.05). Care homes owned by a chain had lower use (odds ratio 0.30; 95% CI 0.14–0.63 versus single ownership care homes), and use was not associated with COVID-19 or staffing levels. Tracker uptake had no impact on controlling COVID-19 spread. Staff self-isolating and local area COVID-19 cases were positively associated with lagged COVID-19 spread in care homes (relative risks 1.29; 1.2–1.4 and 1.05; 1.0–1.1, respectively).

**Conclusions:**

The use of the COVID-19 symptom tracker in care homes was not maintained except in Locality 1 and did not appear to reduce the COVID-19 spread. COVID-19 cases in care homes were mainly driven by care home local-area COVID-19 cases and infections among the staff members. Digital deterioration trackers should be co-produced with care home staff, and local authorities should provide long-term support in their adoption and use.

**Supplementary Information:**

The online version contains supplementary material available at 10.1186/s12879-022-07939-6.

## Background

Residents of care homes (both residential and nursing homes) are particularly vulnerable to developing severe illness from COVID-19 [1]. Over a quarter of all COVID-19-related deaths in England during 2020 were in care homes [2, 3]. By the end of April 2020, a third of all care homes had reported COVID-19 outbreaks [4]. To support care homes, local health and care systems needed real-time data on COVID-19 cases, residents’ health, staff shortages, and availability of personal protective equipment (PPE) to identify those homes, and residents within homes, who needed immediate support to contain and manage COVID-19.

With the emergent threat of COVID-19, a digital tracker was introduced into Greater Manchester (GM) care homes to facilitate proactive management of COVID-19 and residents’ health [5]. The tracker was developed from a digital falls prevention tool^1^ through a partnership between a regional health innovation organisation, a technology company, and clinical leads from one of the localities in GM. The tracker was designed for use on either personal computers, laptops, tablet devices, or other mobile devices for facilitating bedside assessment and input of residents’ health data.^2^

The tracker allowed staff to input residents’ COVID-19-related symptoms (temperature of $37.8^{\circ }$ and/or a new, dry cough) and other health indicators including confusion, health status over the last four weeks, residents’ advance care planning, and whether the resident has been prescribed end of life drugs. Staff members were asked to enter data for each resident to get a snapshot of residents’ health, aid early identification of deterioration, and facilitate care planning and system response.

Real-time data were to be shared with the resident’s general practice (GP) and local NHS community response teams with the hypothesis that this would result in swift measures being put in place (e.g., approaches to manage residents’ health and to help contain COVID-19 spread). Aggregated home-level daily data were visible to area hubs, GPs, and Greater Manchester health and care providers via a visual GM dashboard held by Greater Manchester Health and Social Care Partnership (GMHSCP). This provided a longitudinal dataset of COVID-19 cases and residents’ well-being which included: an interactive map using circles of different colours and sizes to display homes with and without COVID-19 cases, the latest assessment date, daily assessed and reported residents, and a summary dashboard showing trends in COVID-19 and residents’ well-being.

The tracker was deployed in GM, England from April 2020 onward. The deployment was initiated as a partnership between Health Innovation Manchester (HInM), GMHSCP, and a local authority and its NHS Foundation Trust [5]. By the end of 2020, 91 care homes (17% of homes in GM) had adopted the tracker and by April 2021, 139 (25%) of care homes had adopted the tracker. Tracker uptake was mandated for all care homes in one of the ten localities and advised or optional in the remaining nine. In some localities, the tracker was adopted in a small number of care homes to get feedback before wider deployment across the locality. Homes across all localities were expected to do regular assessments (mostly daily) of all the residents for COVID-19 symptoms, confusion signs, and general well-being.

Like many digital technologies deployed worldwide to fight COVID-19 [6], the tracker might affect COVID-19 spread in several ways. The training provided on recording COVID-19 symptoms in the tracker may have improved carers’ understanding of initial COVID-19 symptoms, leading to timely interventions. The sharing of real-time data with GPs and local response teams may have led to prompt interventions such as provision of PPE, addressing staff shortages, providing guidance on keeping distances and self-isolation, and clinical care.

The continuous use and success of such digital trackers depend on a multitude of factors [6–9]. Trackers may have limited success if they are not deemed fit for purpose, useful or where workforce barriers limit their use. They may also be rejected if a lack of system responsiveness to the data is perceived [7, 8]. Similarly, if the tracker was not used in a timely way/effectively, the shared data were not acted upon, or the guidelines were not followed then the tracker might not help in controlling COVID-19 spread or improving any other health indicators in care homes. Additionally, symptom screening alone might not reduce COVID-19 transmission because more than 50% of COVID-19 cases are either mild or occur in asymptomatic residents [6]. On the other hand, if the tracker is thought to be useful for purposes beyond COVID-19 (e.g., recording residents’ health status and advance care plans) then its use might be sustained even if pandemic pressures ease [8].

The evidence published to date on the use and effectiveness of digital technologies to fight COVID-19 has mostly focused on national-level experiences, comparing the use of big data, artificial intelligence, cloud computing, 5G, etc., for remote health services, communication, tracking, and monitoring in the fight against COVID-19 [9–12]. In adult social care, pre-COVID-19 research is mostly qualitative in nature and focused on workers’ and residents’ experiences of digital technologies, barriers and facilitators to their uptake, the application of digital technologies for telehealth, social prescribing, and to support people living with dementia [12, 13]. The post-COVID-19 research has focused on descriptions of interventions, how COVID-19 has changed the prospect of digital technology use, how the new technologies could be harnessed in social care and inequalities in access to digital technologies. The key message from existing published work is that more research is needed to clearly understand the use and effectiveness of such technologies and their impact on digital inequalities [6, 13, 14].

There is limited quantitative evidence on the sustained use of digital trackers, and factors affecting use, for COVID-19 management in adult social care or the impacts such trackers may have on containing the pandemic spread. Particularly, literature on the abandonment of digital innovations versus sustained use is sparse in adult social care [15]. Addressing this is important as uptake alone is not enough for achieving the desired outcomes.^3^

This study aimed to enhance the literature by assessing post-uptake actual use of a digital tracker and whether there was any association between tracker uptake and use and the spread of COVID-19 in care homes in Greater Manchester, UK by answering the following questions:

1. Was there any association between the care home use of a digital COVID-19 tracker and the type of care home, time since tracker adoption, frequency of COVID-19 cases, care home staffing levels, supplies of PPE, and the care home location?
2. Did the uptake and use of a COVID-19 tracker impact on the number of COVID-19 cases in care homes?

## Methods

This study evaluates the use and impact of a digital tracker for COVID-19 management in care homes in Greater Manchester (GM), UK. It is reported according to the STROBE framework [17].

### Intervention

Tracker deployment started in one GM locality in April 2020. Care homes in a second locality began using the tracker in July-August 2020 and by the end of 2020, care homes from eight (of 10) GM localities were using the tracker. In total, 91 GM care homes adopted the tracker at different times during 2020, the number reached 139 by April 2021.

### Study period and population

The study considered all 547 care homes in the GM database across the 10 GM localities. The analysis of tracker use covered the 139 homes that adopted the tracker over the period April 2020 to April 2021. For the tracker impact evaluation, data from year 2021 were excluded due to possible differential effects of COVID-19 vaccination. 13 care homes were dropped from the analysis due to a lack of data on COVID-19 cases. Impact evaluation analysis thus included 534 care homes for the April to December 2020 period.

### Study design and data sources

The study was a prospective cohort analysis of care homes’ use of the tracker and COVID-19 cases in the care homes. Daily data on the number of residents whose health was assessed and reported in the tracker were used to develop a measure of tracker use. These data were obtained via the GM health and care system for the 139 homes that adopted the tracker over the study period. Daily data on care home staffing levels, staff-self-isolating, occupancy levels, number of available beds, residents with COVID-19 (either positive or having symptoms), a flag about whether the home accepts new admissions (yes, limited capacity, emergencies only, not possible) and PPE supplies were provided by the GM health and care system via COVID-19 dashboards for all GM care homes (further details of GM COVID-19 situation reporting data are provided in the Additional file 1: Appendix S1) and linked to data from the Care Quality Commission (CQC) [18] care directory of home types to examine relationships between the tracker use and type of home ownership, residential status (residential vs nursing), quality ratings, and service types (flags on whether the home serves residents with dementia, learning disabilities and/or autism, physical disabilities, and mental health problems, respectively). Middle Super Output Area (MSOA) level COVID-19 weekly cases data were also merged to account for the spread of COVID-19 in the MSOA of the care home. The MSOA-level weekly data were extracted from the publicly available UK COVID-19 dashboard and converted into daily data using geometric growth formula. The data were also merged with data on the home’s local area index of multiple deprivation [19].

### Analysis

The percentage of residents whose health was reported in the tracker was plotted by locality and over time to evaluate geographical and time variations in assessments for the care homes that adopted the tracker. Correlated random effects logistic regression was used to estimate the odds of tracker use (where a home is using the tracker if at least one resident is assessed on a given day) by type of home and across localities [20]. Fixed effects logistic regression tested the association of tracker use with staff levels, COVID-19 cases, PPE availability and change in tracker use with time since adoption. To explore the association between the tracker use and time since adoption, care homes were divided into eight categories; each with a 50 day window. Group 1 comprises care homes adopting the tracker within the last 50 days, and group 8 comprises care homes that adopted the tracker at least 350 days previously. For estimator selection and treatment of missing data, see Additional file 1: Appendix S2.

Correlated random effects logistic regression estimated odds of COVID-19 outbreaks (where an outbreak is defined as having at least one COVID-19 positive resident or resident having symptoms on a given day in a care home) by home residential status, ownership type, CQC quality ratings, and type of residents served. For methods and a discussion of missing data see Additional file 1: Appendix S3.

To assess the impact of the tracker on COVID-19 spread, an event design difference-in-difference (DID) model was estimated [21]. This compared changes in COVID-19 cases per 100 occupied beds (hereafter outcomes) in homes that adopted versus did not adopt the tracker. In an event design, time is measured in relative terms; time to and time since the event of interest. Event design DID is particularly useful in cases where there is variation in timings of intervention implementation across units of interest and a possibility that the effects of intervention might differ across units or over time [22–24]. Given this, the event design DID is recommended for evaluations of COVID-19-related policy interventions [21]. As COVID-19 cases will likely depend not only on tracker use, models were adjusted for lagged values of covariates such as staff available, home local area COVID-19 cases, and a flag on whether the home was accepting new admissions, to reduce potential confounding. Additionally, we used propensity score matching to match the homes that adopted versus did not adopt the tracker by home ownership, residential status, quality ratings, home area deprivation index, and the type of residents served before the DID estimations. The estimations were restricted to 2020 due to possible differential effects of COVID-19 vaccination. 13 care homes were in the database but had no data on COVID-19 or occupied beds during 2020. The DID estimations included 534 care homes with 127,589 observations (homes x days). A range of sensitivity analyses were conducted using Poisson regression and non-parametric estimation techniques (Additional file 1: Appendix S4) [24–26].

## Results

Summary statistics of the number of homes and their key characteristics are provided in Table 1. In GM, 66% of care homes provided only residential services whilst 44% provided nursing or both nursing and residential services. 88% of the care homes were for-profit businesses. 34% of care homes were owned and run by chains whilst 66% were in single ownership. At the beginning of the pandemic, 78% of care homes were rated ‘good’ and 3% were rated ‘outstanding’ by the CQC whilst 18% were rated as ‘requiring improvement’. Care homes that adopted the tracker were larger (by the number of occupied beds and staffing) and owned by a chain than care homes that did not adopt the tracker. There were no statistically significant associations between tracker adoption and most of the other characteristics reported in Table 1.

**Table 1:** Greater Manchester care homes by service type and tracker adoption

	All care homes	Care homes not adopting tracker	Care homes adopting tracker	P-value of difference$^a$
Number of care homes	547	408	139	.
Number of occupied beds per home per day$^b$	28.61	27.45	31.82	0.00
Number of available beds per home per day$^b$	5.20	4.84	6.16	0.00
Number of workers per home per day$^b$	38.57	37.48	41.57	0.00
Number of staff self-isolating per home per day$^b$	0.92	0.84	1.14	0.00
Number of COVID-19 cases per 100 beds per day$^b$	1.95	1.94	1.98	0.36
Care homes with PPE supply of less than a week (%)$^b$	27.12	25.83	30.71	0.27
Care homes with PPE supply of one to four weeks (%)$^b$	67.80	69.05	64.29	0.34
Care homes with PPE supply of more than a month (%)$^b$	5.08	5.12	5.00	.
Care homes with new admissions status: no issue (%)$^b$	41.81	47.06	27.14	0.00
Care homes with new admissions status: limited capacity (%)$^b$	22.79	17.65	37.14	0.00
Care homes with new admissions status: emergencies only (%)$^b$	0.38	0.51	0.00	.
Care homes with new admissions status: not possible (%)$^b$	35.03	34.78	35.71	0.84
Care homes providing residential services only (%)$^c$	66.09	67.37	62.50	0.34
Not-for-profit care homes (%)$^c$	12.02	12.89	9.56	0.36
Branded (chain ownership) care homes (%)$^c$	33.53	29.47	44.85	0.00
Care homes with CQC rating: inadequate (%)$^c$	0.80	0.82	0.74	.
Care homes with CQC rating: requires improvement (%)$^c$	17.37	16.12	20.74	0.23
Care homes with CQC rating: good (%)$^c$	78.44	80.05	74.07	0.18
Care homes with CQC rating: outstanding (%)$^c$	3.39	3.01	4.44	0.41
Care homes with residents with learning disability/autism (%)$^c$	19.38	21.32	13.97	0.08
Care homes with residents with physical disabilities (%)$^c$	37.02	36.32	38.97	0.61
Care homes with residents with mental health issues (%)$^c$	29.07	30.53	25.00	0.27
Care homes with residents with dementia (%)$^c$	54.84	52.89	60.29	0.16

Care homes adopting the tracker are those care homes that adopted the tracker over the April 2020–April 2021 period.

$^a$For the count variables, the p values are from a t-test comparing the difference in means by tracker use. For categorical variables, the p values are from Fisher’s Exact Test on the association between the respective characteristic and the tracker uptake. P values are reported only when $n>30$ for t-test and $n>{5}$ for Fisher Exact test in each group.

$^b$These variables come from the GM COVID-19 situation reporting data. The numbers are mean values over the Apr 2020–Apr 2021 period.

$^c$These variables come from the Care Quality Commission care directory. The percentages are based on 522 care homes as all care homes in the GM COVID-19 situation reporting data could not be linked to the CQC register

### Care home use of the tracker

**Fig. 1:**
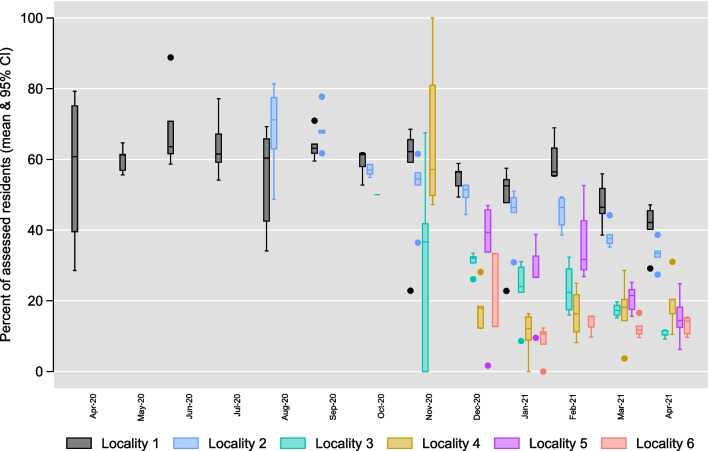
Trends in assessments by local authorities. *CI* confidence interval. The number of care homes that adopted the tracker changes with calender time in each locality. Two localities trends are not reported due to the low number of homes adopting the tracker in those localities

Figure  1 plots the monthly percentage of residents whose health was reported in the tracker across localities and over time. Between April 2020 and April 2021 the average weekly percentage of residents whose health status was reported in the tracker ranged between 13% and 57% across localities (calculated as over time mean of $\frac{weekly assessed}{weekly total}{\times }100$  for each locality). On most days care homes were either reporting every resident (40% days) or none (56% days).

Regression analysis explored variations in tracker use by locality and types of homes (Columns 1 and 2 of Table  2) and examined association with staffing levels, COVID-19 cases and PPE supply (Table  2, Column 3). Since care homes were either reporting all or none of the residents on 96% days, tracker use was measured by a binary indicator taking a value of one if at least one resident was reported on a given day and zero otherwise.

**Table 2:** Associates of post uptake tracker use

	Odd ratio	Odd ratio	Odd ratio
Not-for-profit homes (vs for-profit homes)	0.67 [0.16,2.83]	0.69 [0.17,2.90]	
Residential homes (vs nursing homes)	0.65 [0.31,1.39]	0.76 [0.33,1.76]	
Small homes [1–23 occupied beds]	1.00 [=reference]	1.00 [=reference]	
Medium homes [24–40 occupied beds]	1.20 [0.84,1.72]	1.17 [0.83,1.67]	
Large homes [41+ occupied beds]	1.01 [0.64,1.60]	0.98 [0.62,1.54]	
Chains owned homes (vs single-ownership homes)	0.34 [0.17,0.69]***	0.30 [0.14,0.63]***	
Homes with residents with dementia (yes/no flag)	0.66 [0.28,1.53]	0.51 [0.22,1.15]	
Homes with learning disability and/or autism (yes/no flag)	0.37 [0.11,1.25]	0.38 [0.12,1.27]	
Homes with residents with physical disabilities (yes/no flag)	1.26 [0.64,2.47]	1.23 [0.64,2.35]	
CQC rated inadequate	1.00	1.00	
Requires improvement	0.50 [0.10,2.53]	0.46 [0.09,2.27]	
Good	0.56 [0.13,2.35]	0.45 [0.10,2.04]	
Outstanding	0.33 [0.06,1.78]	0.24 [0.04,1.39]	
Locality 7	1.00	1.00	
Locality 6	0.63 [0.04,9.06]	0.57 [0.04,8.54]	
Locality 5	1.02 [0.12,8.39]	1.15 [0.14,9.74]	
Locality 4	2.44 [0.25,23.74]	1.56 [0.15,15.90]	
Locality 3	2.36 [0.19,29.31]	2.49 [0.20,31.13]	
Locality 2	8.52 [1.00,73.03]*	5.85 [0.67,51.27]	
Locality 1	27.87 [3.26,238.56]***	31.73 [3.76,268.05]***	
Days on tracker [1–49]	1.00	1.00	1.00
Days on tracker [50–99]	0.65 [0.52,0.81]***	0.64 [0.51,0.80]***	0.61 [0.52,0.71]***
Days on tracker [100–149]	0.52 [0.37,0.74]***	0.51 [0.36,0.72]***	0.45 [0.35,0.58]***
Days on tracker [150–199]	0.38 [0.24,0.62]***	0.37 [0.23,0.59]***	0.30 [0.21,0.43]***
Days on tracker [200–249]	0.37 [0.21,0.63]***	0.35 [0.21,0.60]***	0.25 [0.15,0.39]***
Days on tracker [250–299]	0.25 [0.12,0.50]***	0.24 [0.12,0.48]***	0.18 [0.10,0.32]***
Days on tracker [300–349]	0.25 [0.11,0.56]***	0.23 [0.10,0.52]***	0.15 [0.08,0.31]***
Days on tracker [350–399]	0.21 [0.08,0.60]***	0.20 [0.07,0.56]***	0.12 [0.06,0.28]***
Homes COVID-19 +ve residents (%)		0.99 [0.99,1.00]*	0.99 [0.99,1.00]***
Number of workers available for work		0.99 [0.98,1.00]	1.00 [0.99,1.00]
Number of MSOA level COVID-19 cases		1.00 [0.99,1.00]	1.00 [0.99,1.00]
Number of staff self-isolating		0.98 [0.94,1.02]	0.99 [0.96,1.02]
PPE [less than a week]		1.00	1.00
PPE [1 to 4 weeks]		0.59 [0.31,1.13]	0.43 [0.24,0.78]***
PPE [more than 1 month]		0.61 [0.33,1.14]	0.45 [0.24,0.82]***
Accepting new admissions [no issue]		1.00	1.00
Accepting new admissions [limited capacity]		1.03 [0.85,1.26]	1.04 [0.92,1.18]
Accepting new admissions [emergencies only]		1.04 [0.67,1.61]	0.99 [0.53,1.86]
Accepting new admissions [not possible]		1.14 [0.98,1.33]*	1.19 [1.08,1.31]***
Number of occupied beds			1.00 [1.00,1.00]
Home fixed effects			$\checkmark$
Week fixed effects	$\checkmark$	$\checkmark$	$\checkmark$
N	20456	20456	24091
Homes	130	130	137
Chi-Squared	15656082.9	26382872.7	795.3
P-value	0.00	0.00	0.00

Cluster robust 95% confidence intervals are in the brackets. The dependent variable is tracker use [=1 if at least one resident is assessed on a given day in a care home, 0 otherwise]. The coefficient of one locality is not reported due to very low uptake. Columns 1 and 2 exclude 7 homes due to missing CQC data. The estimations in Columns 1 and 2 also include a categorical variable on home local area index of multiple deprivation; the coefficients are mostly insignificant and omitted to save space. The results were obtained from logistic regressions. To prevent omitted variables bias Column 2 includes additional variables and their means (means not reported) . Column 3 removes the time invariant characteristics of homes by including home fixed effects. Details on methods selection and missing data are given in the Additional file 1: Appendix S2. *** p<0.01, ** p<0.05, * p<0.1

Column 1 of Table  2 reports the odds of tracker use by care home types and localities without any adjustment for the available mediating factors such as staffing levels, COVID-19 cases or PPE supply whereas Column 2 adjusts for these factors. From Column 2, the tracker use was 32 times higher (OR 31.73: 95% CI 3.76;268.05) in Locality 1 compared with the reference locality (Locality 7). There was no statistically significant difference in tracker use between all the other localities. Care homes owned by a chain had 70% lower odds of tracker use (OR 0.30: 95% CI 0.14;0.63) compared with independently owned care homes (Column 2 of Table  2).

Column 3 of Table  2 reports the association of staffing levels, COVID-19 cases and PPE supply with tracker use and how use changed since adoption of tracker. Tracker use decreased significantly over time; use was 75% lower after 200 days of adoption compared with the first 50 days of tracker adoption: OR 0.25 (95% CI 0.15;0.39). Tracker use was 57% lower when PPE supply was sufficient for 1 to 4 weeks’ use compared with when PPE supply was sufficient for less than a week (OR 0.43: 95% CI 0.24;0.78). There was only a weak or no association between tracker use and the percentage of residents with COVID-19: 0.99 (95% CI 0.99;1.00) or staff available for work: 1.00 (95% CI 0.99;1.00).

Table A2 in the Additional file 1: Appendix S2 tested the effects of interactions between the time since tracker adoption and local authority indicators by including days on tracker x locality indicators among the regressors. However, most of the coefficients for the interaction terms were statistically insignificant, and the main results of Table  2 still hold. For details on the robustness checks on the factors associated with the tracker use, see the Additional file 1: Appendix S2.

### COVID-19 outbreaks and tracker impact on COVID-19 spread

**Fig. 2:**
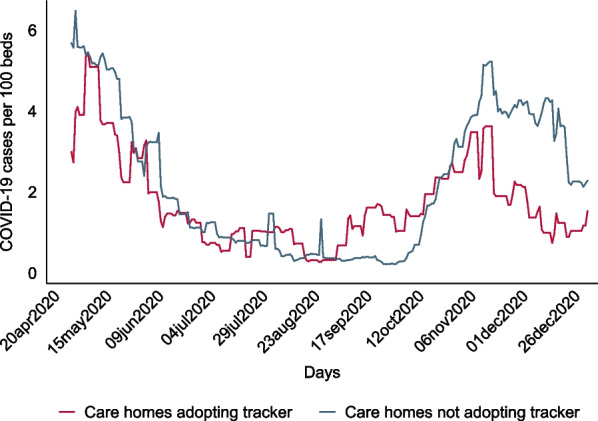
Trends in COVID-19 cases (per 100 beds) by tracker uptake

Figure  2 plots the trends in daily COVID-19 cases per 100 occupied beds in care homes which adopted the tracker versus those which did not adopt the tracker. For both groups, the percentage of COVID-19 positive residents was higher during April, May 2020 and October–December 2020 compared with other months (matching Waves 1 and 2 of the pandemic in England).

Regression analysis explored COVID-19 outbreaks by types of care homes (Columns 1 and 2 of Table  3) and whether adopting the tracker affected COVID-19 spread (Table  3, Columns 3–4). To prevent any effects from differences in COVID-19 vaccination levels the latter analysis was restricted to the year 2020 only.

On 87% of days between April 2020 to April 2021 there was no resident with COVID-19 symptoms or who tested positive. A COVID-19 outbreak was thus measured by a binary indicator taking a value of one if at least one resident tested positive or had symptoms on a given day, zero otherwise. Column 1 of Table  3 reports the odds of outbreaks by care home types without any adjustment for time variant mediating factors whereas Column 2 adjusts for the available mediating factors.

From Column 2, the odds of experiencing a COVID-19 outbreak were lower in larger care homes [41+ occupied beds] (OR 0.54: 95% CI 0.30;0.98) compared with small homes [1–23 occupied beds] and care homes with residents who had learning disabilities and/or autism (OR 0.18: 95% CI 0.09;0.34) compared with care home with no such residents. On the other hand, care homes CQC rated as requiring improvement and care homes owned by a chain had higher odds of COVID-19 outbreaks (ORs 6.14: 95% CI 1.13;33.42 and 1.44: 95% CI 1.02;2.04, respectively). Not-for-profit care homes and homes with dementia users experienced more outbreaks and residential only care homes fewer outbreaks but the difference became insignificant after controlling for staff, home local area cases, and PPE. This means that the differences in the odds of COVID-19 outbreaks in these latter care homes were mediated by differences in their staff, home local area cases or PPE supplies.

**Table 3:** COVID-19 outbreaks by types of care homes and the impact of the tracker in COVID-19 containment

	Outbreaks by home types		Tracker impact		Other drivers
	Odd ratio	Odd ratio	Relative risk	Relative risk	Relative risk
Not-for-profit homes (vs for-profit homes)	2.46 [1.39,4.37]***	1.49 [0.90,2.47]			
Residential homes (vs nursing homes)	0.38 [0.25,0.59]***	0.83 [0.56,1.22]			
Small homes [1–23 occupied beds]	1.00 [=reference]	1.00 [=reference]			
Medium homes [24–40 occupied beds]	0.76 [0.51,1.14]	0.82 [0.54,1.25]			
Large homes [41+ occupied beds]	0.48 [0.27,0.86]**	0.54 [0.30,0.98]**			
Chains owned homes (vs single-ownership homes)	2.09 [1.44,3.05]***	1.44 [1.02,2.04]**			
Homes with residents with dementia (yes/no flag)	1.89 [1.26,2.83]***	1.21 [0.85,1.74]			
Homes with learning disability and/or autism (yes/no flag)	0.19 [0.10,0.38]***	0.18 [0.09,0.34]***			
Homes with residents with physical disabilities (yes/no flag)	1.08 [0.74,1.60]	1.17 [0.82,1.67]			
Inadequate CQC rated	1.00	1.00			
Requires improvement	6.00 [0.86,42.04]*	6.14 [1.13,33.42]**			
Good	3.63 [0.52,25.19]	4.42 [0.82,23.95]*			
Outstanding	4.01 [0.47,34.04]	2.71 [0.40,18.29]			
Tracker uptake lag 7 (yes/no flag)	1.31 [0.81,2.13]	1.39 [0.87,2.22]	0.72 [0.42,1.25]	0.60 [0.35,1.04]*	0.72 [0.45,1.13]
Number of MSOA level COVID-19 cases lag 7		1.01 [1.00,1.02]***		1.06 [1.04,1.08]***	1.05 [1.03,1.07]***
Number of workers available lag 7		0.99 [0.98,1.00]		0.98 [0.97,0.99]***	0.99 [0.98,1.00]
Accepting new admissions [no issue] lag 7		1.00		1.00	1.00
Accepting new admissions [limited capacity] lag 7		1.38 [1.05,1.82]**		1.25 [0.85,1.83]	1.21 [0.83,1.76]
Accepting new admissions [emergencies only] lag 7		2.61 [1.21,5.62]**		0.86 [0.31,2.41]	0.90 [0.44,1.83]
Accepting new admissions [not possible] lag 7		2.01 [1.56,2.58]***		1.72 [1.19,2.50]***	1.56 [1.10,2.21]**
Worker RAG [Green] lag 7		1.00		1.00	1.00
Worker RAG [Amber] lag 7		1.43 [1.01,2.03]**		2.11 [1.68,2.66]***	1.18 [0.92,1.51]
Worker RAG [Red] lag 7		2.58 [1.39,4.79]***		4.28 [3.07,5.97]***	1.41 [0.99,2.01]*
Number of staff self-isolating lag 7		1.18 [1.11,1.25]***			1.29 [1.22,1.36]***
PPE [less than a week] lag 7		1.00			1 .00
PPE [1 to 4 weeks] lag 7		0.71 [0.49,1.02]*			0.74 [0.55,0.99]**
PPE [more than 1 month] lag 7		0.82 [0.49,1.39]			0.88 [0.48,1.61]
Number of beds available lag 7				1.01 [1.00,1.02]	1.00 [0.99,1.02]
Number of occupied beds lag 7				0.98 [0.97,1.00]**	0.99 [0.97,1.00]**
Home random effects	$\checkmark$	$\checkmark$			
Home fixed effects			$\checkmark$	$\checkmark$	$\checkmark$
LA fixed effects	$\checkmark$	$\checkmark$	$\checkmark$	$\checkmark$	$\checkmark$
Week fixed effects	$\checkmark$	$\checkmark$	$\checkmark$	$\checkmark$	$\checkmark$
N	161,999	159,492	95,836	95,033	95,026
Homes	497	497			
Chi-Squared	665.1 (p-val=0.00)	923.2 (p-val=0.00)			
Pseudo R-Squared			0.318	0.380	0.433
Homes adopting tracker (estimation sample)			83	83	83
Homes not adopting tracker (estimation sample)			327	327	327

95% clustered robust confidence intervals are in the brackets. The dependent variable in Columns 1 and 2 is COVID-19 outbreaks [=1 if at least one resident has COVID-19 symptoms or tested positive on a given day, 0 otherwise]. The dependent variable in Columns 3–5 is COVID-19 symptomatic/positive residents per 100 beds. Column 1 estimated with random effect logistic, Column 2 with correlated random effects logistic models and Columns 3–5 with fixed effects Poisson regression (coefficients are incidence rate ratios). The estimations in Columns 1 and 2 also include a categorical variable on home local area index of multiple deprivation; the coefficients are mostly insignificant and omitted to save space. Column 4 does not control for any confounding factors. Column 5 includes factors other than PPE and staff self-isolating as these might be caused by the tracker uptake. All the control variables are included with lag 7 keeping in view the incubation period of COVID-19. Details on methods selection, missing data, and further sensitivity analysis are given in the Additional file 1: Appendices S3 and S4. *** p<0.01, ** p<0.05, * p<0.1

Several parametric and non-parametric DID estimations were run to assess the impact of the tracker on COVID-19 cases among care residents. No statistically significant difference was found in the number of COVID-19 cases between the care homes that did and did not adopt the tracker. The unadjusted and adjusted estimation results for the standard DID are given in Table  3, Columns 3 and 4 (see the coefficient of tracker uptake lag 7). The event design DID estimations summary is provided in Table  4. Except for some random instances, none of the pre-tracker’s uptake and post-tracker uptake coefficients are statistically significant in Table  4. This finding is particularly clear after adjusting for all available covariates (Column 4 of Table 4). This implies that, after adjusting for the covariates, adoption of the tracker did not appear to influence rates of COVID-19. Results of additional sensitivity analyses, including parametric and non-parametric estimations (with and without matching on care home types and quality ratings) for all care homes and sub sample of homes from only 4 GM localities are reported in the Additional file 1: Appendix S4.

Our analyses identified several other factors that were consistently associated with the number of COVID-19 cases in care homes (Table  3, Column 5). The number of staff self-isolating and the number of COVID-19 cases in the MSOA area where the home was situated were positively associated with COVID-19 cases in care homes. From Table  3, one additional staff member self-isolating was associated with a 29% increase in care home COVID-19 cases per 100 occupied beds seven days later (RI 1.29 :95% CI 1.22; 1.36). Similarly, there was a 5% increase in care home COVID-19 cases per 100 occupied beds for each additional COVID-19 confirmed case in the home MSOA area: RI 1.05 (95% CI 1.03; 1.07). Care homes not accepting new residents had 56% more COVID-19 cases per 100 beds seven days later compared with care homes accepting new residents (RI 1.56 (95% CI 1.10; 2.21)). But this does not necessarily mean that issues in accepting new residents into homes caused COVID-19, indeed many care homes reported COVID-19 outbreak as a reason for not accepting new residents.

Sufficient PPE to last 1 to 4 weeks was associated with 26% fewer COVID-19 cases per 100 beds seven days later compared with PPE supply that was sufficient for less than a week: RI 0.74 (95% CI 0.55; 0.99). However, this result does not hold in the additional sensitivity analyses in the Additional file 1: Appendix S4. Finally, the number of workers available for work had no association with COVID-19 cases.

**Table 4:** DID estimates of COVID-19 cases per 100 beds in the pre and post tracker use periods

	Unadjusted event design	Adjusted event design	Matching based event design	Adjusted event design
Prior 34th day	1.24	1.50	3.22**	1.33
	[0.54,2.84]	[0.63,3.56]	[1.15,9.01]	[0.60,2.95]
28 days to event	1.16	1.07	1.10	1.23
	[0.34,3.93]	[0.31,3.74]	[0.22,5.58]	[0.38,3.98]
21 days to event	1.03	1.05	1.21	1.09
	[0.34,3.07]	[0.38,2.90]	[0.54,2.71]	[0.39,3.03]
14 days to event	1.53	1.49	1.30	1.57
	[0.65,3.58]	[0.68,3.27]	[0.58,2.92]	[0.68,3.60]
7 days to event	1.44	1.39	1.59	1.39
	[0.88,2.37]	[0.86,2.25]	[0.79,3.19]	[0.94,2.06]
1 day to event	1.18	1.35	1.77	1.36
	[0.76,1.83]	[0.82,2.20]	[0.82,3.83]	[0.86,2.17]
1 day after event	0.80	0.83	1.07***	0.84
	[0.53,1.21]	[0.51,1.37]	[1.03,1.11]	[0.51,1.38]
7 days after event	0.68	0.70	0.82	0.70
	[0.37,1.26]	[0.35,1.39]	[0.47,1.44]	[0.32,1.53]
14 days after event	0.40**	0.38**	0.51	0.45*
	[0.19,0.85]	[0.17,0.86]	[0.19,1.42]	[0.20,1.02]
21 days after event	0.83	0.73	0.55	0.90
	[0.24,2.87]	[0.19,2.75]	[0.13,2.28]	[0.24,3.32]
28 days after event	1.10	0.78	1.26	0.85
	[0.27,4.48]	[0.19,3.13]	[0.37,4.32]	[0.22,3.31]
Beyond 34th day	1.08	1.05	1.67	1.03
	[0.43,2.75]	[0.39,2.81]	[0.56,5.00]	[0.44,2.41]
N	98,890	95,033	41,245	95,026
Homes	410	406	174	406
Pseudo R-squared	0.314	0.381	0.394	0.434

Cluster robust 95% confidence intervals are in the brackets. The coefficients are incidence rate ratios. For additional covariates included in each of the columns, see Table A3 in the Additional file 1: Appendix S4. Column 3 estimations were run on a matched sub-sample (matching was done on all the characteristics reported in the CQC data given in Table 1 with nearest 5 neighbours for each treated home). The estimations included 34 pre and 34 post uptake event indicators, but to save space reports only the ones with seven days gap. ***p<0.01, **p<0.05, *p<0.1

## Discussion

The onset of COVID-19 led to a host of digital innovations to fight the pandemic. Digital technology has played a key role alongside physical restrictions in containing the virus, and in the provision of online health and other social-economic services [10, 11]. Literature on the use of digital technologies during COVID-19 has focused on: the types of digital technologies used and the way they were used; the challenges use of these technologies created; their effectiveness in tracking COVID-19 spread; and how these technologies can be integrated into the health and care systems [10, 11]. To date, adult social care related studies have mostly focused on the role of such technologies in telemedicine, and the digital divide COVID-19 has created for the elderly [12, 13]. One key concern about digital innovations in health and social care is the abandonment of such technologies even after initial uptake [15, 16].

This study contributes to the existing knowledge by analysing the use of a digital COVID-19 tracker over time and space, the factors associated with use and its impact on the spread of COVID-19. The study found that among the 139 care homes that adopted the tracker, its use decreased by more than 75% within one year of deployment. Tracker use was 30 times higher in care homes in the locality where the tracker was developed and tested. Tracker use was 66% lower in care homes owned and run by chains than in individually owned homes. Moreover, tracker use was negatively associated with care home PPE supplies and was not associated with COVID-19 rates or staffing levels.

The study analysed whether the odds of experiencing COVID-19 outbreaks were associated with any specific care home characteristics. The findings suggested that the odds of experiencing COVID-19 outbreaks were higher in care homes owned by a chain (odds of the outbreak were twice compared with single-ownership care homes) and homes rated as requiring improvement. The odds of experiencing COVID-19 outbreaks were 80% lower in care homes with residents with learning disabilities and/or autism.

The final part of the study analysed whether the tracker had any impact on rates of COVID-19 in care homes and found no difference in the rates of COVID-19 according to whether homes adopted the tracker or not. The two main drivers of the rates of COVID-19 in care homes appeared to be COVID-19 cases in the local area and amongst the care home staff. A staff member self-isolating on a given day was associated with a 29% increase in COVID-19 cases among the care residents seven days later. The findings that digital symptom tracking had no impact on containing COVID-19 spread in care homes, and that workers and home surroundings were key drivers of COVID-19 spread into care homes, are also confirmed in other studies [27–29].

The finding that the odds of tracker use were higher in Locality 1 than the reference Locality 7 and that the percentage of residents receiving daily assessments was also relatively stable overtime (Fig.  1) in Locality 1 compared with other localities, has important implications. The local authority and associated health and care system in Locality 1 were partners in the development and initial deployment of the tracker [5]. This finding resonates with qualitative work that has found local production and support as the key success factors in digital technologies adoption in adult social care and elsewhere [6, 30, 31]. Additionally, the concurrent implementation study [30] of the tracker compared implementation in four of the localities and found health and care systems were digitally well integrated in Locality 1, compared with the other three localities. Thus, the higher tracker use in Locality 1 is a reflection of better systems of data flows, better response to the data created needs, digital literacy and culture of digital applications among care staff, or strong local council leadership that encourages digital innovations [6, 30, 31].

The pressure the COVID-19 pandemic placed on adult social care was one reason for lower uptake and use of the tracker as recognised in the parallel implementation study [30]. Nevertheless, the decline in tracker use over time and the lack of consistent association of use with COVID-19 cases suggests that care homes may have felt the tracker was unnecessary, perhaps due to the development of testing capacity, vaccination, and better PPE supply [30]. However, the assessment and reporting of residents’ well-being was a key component of the tracker and one could assume that homes would continue its use if they felt it helped promote residents’ health by encouraging regular assessment, active intervention and support from the local health systems. Our data did not suggest a concomitant reduction in the percentage of residents who were unwell over the study period and therefore the decline in tracker use suggests that homes in most localities did not feel that it invoked a useful system response.

The finding that the tracker was ineffective in controlling COVID-19 spread in care homes is plausible for two reasons. Firstly, it has been shown that 50 to 70% of care home residents with COVID-19 were asymptomatic [6, 29], so a symptom tracker could only ever identify a proportion of COVID-positive residents. Secondly, the low frequency of assessments and possible lack of active response from the local health and care system might be another reason for the lack of apparent impact of the tracker on spread of COVID-19.

This study has several strengths. The study is particularly novel in its assessment of tracker use, enabling an assessment of use over time since adoption that adjusted for a range of potential confounding factors. The impact assessment also adjusted for a range of potential mediating factors with consistent findings across a range of sensitivity analyses. Secondly, our analyses examined COVID-19 spread in care homes within a region with its own devolved health and care system [32]. The local focus controls for regional policy variations and facilitated the ability to observe use and impact with local-level data being available that may be more difficult to observe in national initiatives. Thirdly, the study findings are complemented by a contemporaneous qualitative study explaining the factors that facilitated/inhibited the implementation and use of the tracker [30].

However, this was an observational study and has limitations. The study used a combination of COVID symptoms data and confirmed cases of COVID from the GM COVID-19 situation report instead of confirmed COVID cases only for the impact evaluation. These have both merits and weaknesses. To use confirmed cases data, one must know the testing regimes of homes as low/high COVID-19 cases among homes might be the reflection of differences in testing regimes. Conversely data based on symptoms will miss asymptomatic residents and potentially misclassify people has having COVID-19 when they have symptoms due to other illnesses. Whilst symptoms may not necessarily represent confirmed COVID-19 cases, we found a close association between our COVID-19 data and COVID-19 related deaths in care homes (see the Additional file 1: Appendix S1).

The data used for the COVID-19 impact evaluation of the tracker were an unbalanced panel as some care homes started data submission at later stages of the study period. Unbalanced panels always raise the possibility of selection bias. Our inspection of data revealed that care homes from different local authorities started submission at different calendar dates. Thus, it is unlikely that any unobserved care home characteristic was the reason for the data being an unbalanced panel. Similarly, the initial uptake of the tracker was largely decided by local authorities. Nevertheless, the descriptive statistics revealed that care homes that adopted the tracker were larger on average, raising the possibility of selection into the tracker. To account for the possible selection bias, the sensitivity analyses matched the care homes on different CQC characteristics before the estimation of tracker impact. Despite all these, the study finding of no causal association between tracker uptake and COVID-19 spread should be taken with caution.

The study also analysed the association of staff infections and local area COVID-19 cases with COVID-19 spread in care homes. COVID-19 cases in care home residents might come from COVID-19 infections among the care home staff and local area COVID-19 cases and vice versa. However, exploring both possibilities were not the focus of this study. The current study only explored the relationship between infection of a member of care home staff and resident COVID-19 cases seven days later.

Our study suggests several potential areas for future exploration. First, though, the tracker was ineffective in controlling the virus spread, such trackers might have impacted on the number of residents admitted to hospital and/or COVID-19-related deaths through active information sharing and responses from the local health systems. One possible area of investigation might be the impact of such trackers on excess deaths in care homes. Similarly, the tracker might have impacted the quality of residents’ care or had workforce implications. The digital skills the adult social care workforce developed during the pandemic might have enhanced their capabilities and motivation to co-produce and evaluate digital solutions for online consultations, falls prevention, and early signs of deterioration. Finally, the relationship between homes owned by a chain, tracker use, and COVID-19 outbreaks needs further study to understand the mechanisms behind this, as similar findings emerged from a study in Canada as well [33].

## Conclusion

This study demonstrated that the post-uptake use of a digital tracker deployed in GM care homes during COVID-19 was short-lived except in a local authority that was a partner in its initial development and deployment and has a relatively well-integrated health and social care system. COVID-19 cases in care homes were mainly driven by care home local-area COVID-19 cases and infections among the staff members, and the digital symptom tracking was not helping in containing COVID-19 spread in care homes.

COVID-19 has driven the development and adoption of new digital technologies at scale and speed. During COVID-19 the focus was on the uptake of digital technologies and removing barriers to uptake. The learning from the initiatives during COVID-19 may be used to identify practices that can be put in place for creating a supportive environment for sustained and effective use of digital technologies. The findings from this study imply that strong local leadership and co-production might be key success factors in the sustained use of digital technologies in adult social care. Similarly, the integration of primary, secondary, and social care systems are identified as enablers in increasing engagement with digital technologies [30]. Clear evidence of their effectiveness shall help to convince wider adoption and sustained use.

## Supplementary Information

**Additional file 1: Appendix S1.** Explains the dataset. **Appendix S2.** Discusses the estimation methods and sensitivity analysis tables of the tracker use. **Appendix S3.** Outlines the estimation method selection for estimating the odds of COVID-19 outbreaks. **Appendix S4.** Contains details of estimation methods and sensitivity analysis tables for the tracker impact on COVID-19 spread.

## Data Availability

The data that support the findings of this study are owned by the Greater Manchester Health and Social Care Partnership and available only upon reasonable request and signed data access agreement. Data are however available from the authors upon reasonable request and with permission of the Greater Manchester Health and Social Care Partnership.

## Abbreviations

CI: Confidence interval
CQC: Care Quality Commission
DID: Difference-in-difference
GM: Greater Manchester
GMHSCP: Greater Manchester Health and Social Care Partnership
GP: General practice
HInM: Health Innovation Manchester
MSOA: Middle super output area
NHS: National Health Service
PPE: Personal protective equipment
UK: United Kingdom

## Footnotes

1 For a general introduction of Falls app and COVID tracker, see the company site https://safesteps.tech/covid-tracker.

2 For a brief introduction and template of the tracker deployed in GM, see the user guide https://healthinnovationmanchester.com/wp-content/uploads/2020/07/CV19-Care-Home-Dashboard-User-Guide-Jul20.pdf.

3 For example, in Norway twenty-one out of 28 hospitals reported using telemedicine, however relative use of telemedicine compared to that of outpatient visits in qualifying specialties was only about one percent [16].
