# Supplementary material for: The use and impact of digital COVID-19 tracking in adult social care: a prospective cohort study of care homes in Greater Manchester
Source: BMC Infect Dis. 2023 Jan 23;23:47. doi: 10.1186/s12879-022-07939-6 (PMC9869837; doi:10.1186/s12879-022-07939-6)
Supplement: Supplementary file 1 — Additional file 1: Appendix S1. Explains the dataset. Appendix S2. Discusses the estimation methods and sensitivity analysis tables of the tracker use. Appendix S3. Outlines the estimation method selection for estimating the odds of COVID-19 outbreaks. Appendix S4. Contains details of estimation methods and sensitivity analysis tables for the tracker impact on COVID-19 spread. [file 12879_2022_7939_MOESM1_ESM.pdf]

The use and impact of digital COVID-19 tracking in adult social care: a prospective cohort study of care homes in Greater Manchester

Additional File 1

Appendix S1

GM COVID situation reporting dataset

From 20th April 2020, care homes across the ten local authorities in GM submitted daily data into GM COVID-19 dashboard maintained by GMHSCP. The data were sourced from the GM situation reporting (SitRep) submitted by each local authority across GM. These data were collected for monitoring the COVID-19 situation and were independent of homes' tracker use. Most of the homes started submissions from the last week of April 2020, with few initiating submissions at earlier/later dates (hence an unbalanced panel of 547 homes). The daily submission rate (out of total GM care homes) was more than 90% between April-December 2020.

COVID-19 outcomes The dependent variable for the impact evaluation of the tracker was residents with positive COVID-19 and/or those with symptoms per 100 occupied beds. After June 2020, most of the residents with COVID-19 were those who tested positive, but data in the earlier stage of COVID-19 predominantly included residents with symptoms as the testing capacity was not developed at this stage. To assess reliability of data, we aggregated daily COVID-19 data from the GM COVID situation reporting to the local authority level and plotted it along with COVID-19 related deaths of care homes residents in the local authority. Figure A1 gives a very close association between the GM SitRep COVID-19 positive/symptoms data and the care homes COVID-19 related deaths from the Office of National Statistics.

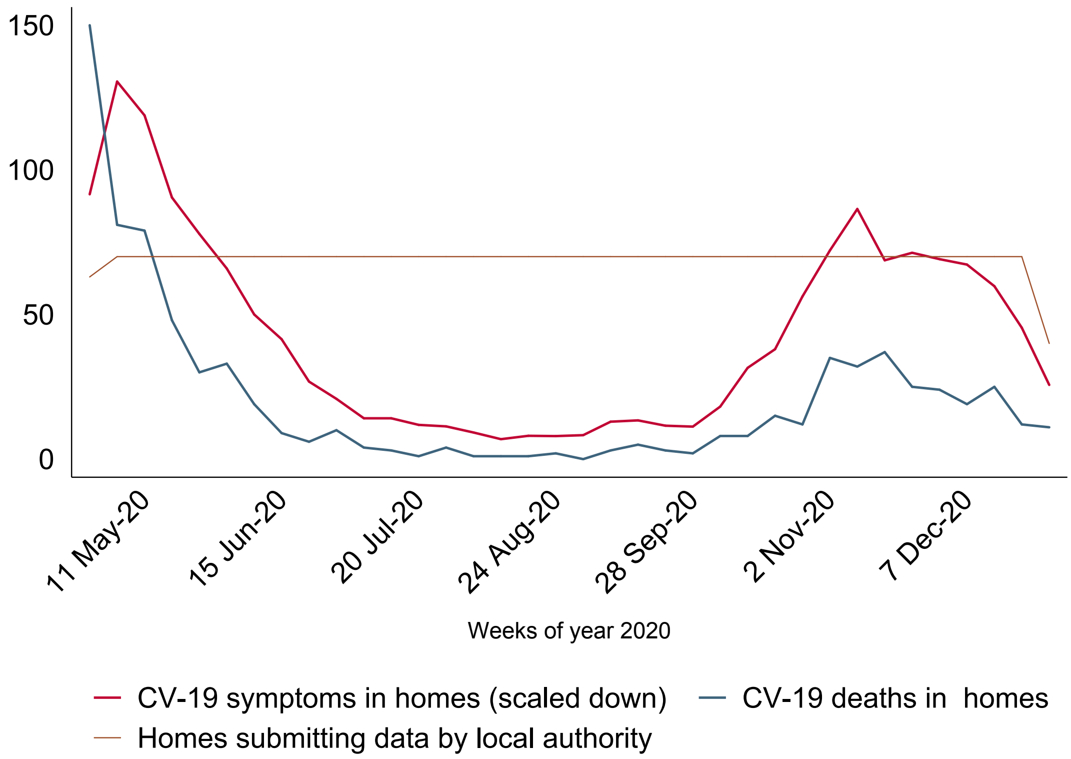

Figure A1: Greater Manchester care homes COVID-19 cases and deaths trends

Appendix S2

Estimation methods and sensitivity analysis tables of the tracker use

Tracker use To assess the factors affecting care homes use of tracker, the binary indicator of use was regressed on a number of variables along with weeks, local authority and home dummies for the 139 care homes (manuscript: Table 2). Three regression models were estimated: 1) Random effects logistic, 2) Correlated random effects logistic, and 3) Fixed effects logistic regression.

Fixed effects model provides consistent estimates even if there are observed or unobserved time invariant characteristics of care homes that are correlated with the observed variables like staffing levels, PPE, and occupied beds. The model includes a dummy variable for each home that captures the overall effects of all such observed/unobserved time invariant characteristics of care home. A disadvantage of the fixed effects model is thus that it cannot retrieve the separate effects of the

time invariant variables, such as the effects of homes residential, legal and ownership statuses that remain constant over time. On the other hand, random effects model assumes that unobserved time invariant characteristics of homes must not correlate with the covariates that are included in the regression model.

An intermediate approach that helps retrieve the effects of the observed time invariant characteristics and relies on a weaker assumption than the random effects model is the correlated random effects model.[1] This model separates within- and between- home effects by including the original variables and their home specific mean values for the variables that vary across homes and over time (such as staffing levels, PPE, and occupied beds). Thus, when our interest was to retrieve the effects of localities and home types, we used correlated random effects regressions (manuscript: Column 2 of Table 2).

Missing data Two of the homes adopted the tracker in the last week of the sample and were dropped from the analysis. Seven care homes were dropped from the correlated random effects regression as those could not be linked to CQC data on homes' characteristics. Rest of the data were complete except for the use indicator, having approximately 4% missing values. This means that missing data were highly unlikely to create any bias in our results.[2] As a further check, the missingness in use indicator was regressed on the observed covariates along with care home fixed effects. None of the covariates were associated with the missing observations in the use indicator after controlling for home fixed effects. This implies that the missing data in the use indicator is missing completely at random (MCAR), and complete case analysis is a valid approach.[3]

The results in Table A1 replaced the time since adoption indicators of Table 2 with calendar month indicators and indicators on the number of care homes with the tracker on a given day. The former indicators capture how the tracker use decreases over calendar time, whereas the latter indicators control for the number of care

[1] Wooldridge, J. M. (2019). Correlated random effects models with unbalanced panels. Journal of Econometrics, 211(1), 137-150.

[2] Schafer, J. L. (1999). Multiple imputation: a primer. Statistical methods in medical research, 8(1), 3-15.

[3] Faria, R., Gomes, M., Epstein, D., and White, I. R. (2014). A guide to handling missing data in cost-effectiveness analysis conducted within randomised controlled trials. Pharmacoeconomics, 32(12), 1157-1170: (https://doi.org/10.1007/s40273-014-0193-3).

Table A1: Associates of post uptake tracker use (An alternative specification)

	Odd ratio	Odd ratio	Odd ratio	Odd ratio
Not-for-profit homes (vs for-profit homes)	0.73 [0.18,2.99]	0.75 [0.19,3.01]		
Residential homes (vs nursing homes)	0.78 [0.37,1.64]	0.92 [0.40,2.10]		
Small homes [1–23 occupied beds]	1.00 [=reference]	1.00 [=reference]		
Medium homes [24–40 occupied beds]	1.17 [0.84,1.64]	1.15 [0.83,1.59]		
Large homes [41+ occupied beds]	0.98 [0.63,1.54]	0.95 [0.61,1.47]		
Chains owned homes (vs independent homes)	0.33 [0.16,0.66]***	0.27 [0.13,0.57]***		
Homes with residents with dementia (yes/no flag)	0.60 [0.27,1.35]	0.49 [0.23,1.07]*		
Homes with learning disability and/or autism (yes/no)	0.48 [0.15,1.54]	0.49 [0.15,1.56]		
Homes with residents with physical disabilities (yes/no)	1.37 [0.72,2.58]	1.30 [0.70,2.42]		
CQC rated inadequate	1.00	1.00		
Requires improvement	0.49 [0.10,2.50]	0.42 [0.08,2.11]		
Good	0.50 [0.12,2.09]	0.39 [0.09,1.75]		
Outstanding	0.28 [0.05,1.51]	0.20 [0.03,1.16]*		
Locality 7	1.00	1.00		
Locality 6	2.24 [0.23,22.35]	1.50 [0.14,15.89]		
Locality 5	1.01 [0.12,8.59]	1.19 [0.13,10.61]		
Locality 4	0.55 [0.04,7.85]	0.54 [0.04,7.75]		
Locality 3	2.67 [0.21,33.48]	2.68 [0.21,34.63]		
Locality 2	5.58 [0.63,49.28]	4.50 [0.49,41.13]		
Locality 1	10.29 [1.22,86.91]**	11.60 [1.39,96.74]**		
Apr-20	1.00	1.00	1.00	1.00
May-20	0.26 [0.08,0.85]**	0.28 [0.08,0.90]**	0.27 [0.10,0.74]**	0.33 [0.14,0.76]***
Jun-20	64.25 [6.90,598.30]***	66.69 [6.92,642.44]***	25.41 [4.71,137.02]***	0.38 [0.15,0.93]**
Jul-20	3.63 [0.83,15.96]*	3.81 [0.85,17.15]*	2.73 [0.73,10.17]	0.31 [0.13,0.75]***
Aug-20	0.36 [0.09,1.40]	0.37 [0.10,1.45]	0.30 [0.10,0.91]**	0.17 [0.07,0.44]***
Sep-20	0.84 [0.19,3.72]	0.88 [0.20,3.90]	0.72 [0.19,2.75]	0.15 [0.06,0.39]***
Oct-20	0.28 [0.06,1.19]*	0.28 [0.07,1.24]*	0.24 [0.06,1.02]*	0.13 [0.05,0.34]***
Nov-20	0.05 [0.01,0.25]***	0.05 [0.01,0.26]***	0.04 [0.01,0.20]***	0.15 [0.06,0.39]***
Dec-20	0.02 [0.00,0.12]***	0.02 [0.00,0.13]***	0.02 [0.00,0.09]***	0.15 [0.06,0.41]***
Jan-21	0.02 [0.00,0.12]***	0.02 [0.00,0.12]***	0.01 [0.00,0.06]***	0.13 [0.05,0.34]***
Fab-21	0.08 [0.01,0.47]***	0.08 [0.01,0.48]***	0.04 [0.01,0.23]***	0.29 [0.10,0.79]**
Mar-21	0.20 [0.03,1.21]*	0.20 [0.03,1.27]*	0.14 [0.02,0.90]**	0.23 [0.08,0.65]***
Apr-21	0.06 [0.01,0.38]***	0.06 [0.01,0.40]***	0.04 [0.01,0.25]***	0.18 [0.07,0.52]***
Number of homes on a given day [1–19]	1.00	1.00	1.00	1.00
Number of homes on a given day [20–39]	0.73 [0.30,1.77]	0.72 [0.30,1.75]	0.94 [0.45,1.96]	1.23 [0.86,1.77]
Number of homes on a given day [40–59]	74.84 [11.70,478.50]***	75.36 [11.39,498.82]***	149.59 [27.32,819.08]***	2.29 [1.44,3.64]***
Number of homes on a given day [60–79]	29.66 [5.45,161.38]***	29.64 [5.28,166.45]***	57.58 [11.52,287.88]***	2.16 [1.29,3.63]***
Number of homes on a given day [80–99]	8.87 [1.74,45.07]***	8.77 [1.68,45.90]**	19.31 [4.08,91.39]***	1.57 [0.90,2.75]
Number of homes on a given day[100–119]	8.08 [1.51,43.37]**	7.96 [1.45,43.81]**	17.43 [3.60,84.39]***	1.42 [0.78,2.58]
Number of homes on a given day [120–139]	2.00 [0.39,10.43]	1.97 [0.37,10.49]	4.51 [0.89,22.78]*	0.59 [0.31,1.12]
Homes COVID-19 +ve residents (%)		0.99 [0.98,1.00]**	0.99 [0.98,0.99]***	0.99 [0.98,0.99]***
Number of workers available for work		0.99 [0.98,1.00]*	0.99 [0.98,1.00]*	0.99 [0.99,1.00]*
Number of MSOA level COVID-19 cases		1.00 [0.99,1.01]	1.00 [0.99,1.00]	1.00 [1.00,1.00]
Number of staff self-isolating		0.98 [0.94,1.03]	0.99 [0.96,1.02]	0.99 [0.96,1.02]
PPE [Less than a week]		1.00	1.00	1.00
PPE [1 to 4 weeks]		0.59 [0.32,1.10]*	0.49 [0.27,0.89]**	0.49 [0.27,0.89]**
PPE [More than 1 month]		0.62 [0.34,1.13]	0.52 [0.28,0.96]**	0.53 [0.29,0.98]**
Accepting new admissions [No issues]		1.00	1.00	1.00
Accepting new admissions [Limited Capacity]		1.01 [0.82,1.24]	1.00 [0.88,1.14]	1.00 [0.89,1.14]
Accepting new admissions [Emergencies only]		1.02 [0.69,1.51]	0.98 [0.52,1.84]	0.93 [0.50,1.76]
Accepting new admissions [Not Possible]		1.13 [0.97,1.33]	1.17 [1.07,1.29]***	1.16 [1.05,1.27]***
Number of occupied beds			1.00 [0.99,1.00]	1.00 [1.00,1.00]
Home fixed effects			✓	✓
Week fixed effects	✓	✓	✓	
N	20724	20724	24040	24040
Homes	132	132	139	139
Chi-Squared	17064326.8	22365568.3	1000.8	669.7
P value	0.00	0.00	0.00	0.00

Notes: *** p<0.01, ** p<0.05, * p<0.1. Cluster robust 95% confidence intervals are in the brackets. The dependent variable is tracker use [=1 if at least one resident is assessed on a given day in a care home, 0 otherwise]. The coefficient of one locality is not reported due to very low uptake. Columns 1 and 2 exclude 7 homes due to missing CQC data. The estimations in Columns 1 and 2 also include a categorical variable on home local area index of multiple deprivation; the coefficients are mostly insignificant and omitted to save space. The results were obtained from logistic regressions. To prevent omitted variables bias Column 2 includes additional variables and their means (means not reported) . Columns 3 and 4 removes the time invariant characteristics of homes by including home fixed effects.

homes on a given day to account for the differences in use that might arise from new homes adopting the tracker. These results also confirmed that tracker use decreased over time, care homes owned by a chain had lower odds of tracker use, and use was significantly higher in Locality 1 compared with the other localities, though the magnitude of the difference is now lower compared with Table 2. Table A2 introduced interactions between the time since adoption and locality indicators to access whether the main results of Table 2 hold after further generalisation of the regression model.

Table A2: Associates of post uptake tracker use (with interaction terms)

	Odd ratio	Odd ratio
Days on tracker [1–49]	1.00 [=reference]	1.00 [=reference]
Days on tracker [50–99]	0.46 [0.27,0.79]***	0.46 [0.26,0.80]***
Days on tracker [100–149]	0.76 [0.43,1.34]	0.73 [0.40,1.32]
Days on tracker [150–199]	0.45 [0.24,0.84]**	0.44 [0.24,0.82]***
Days on tracker [200–249]	0.36 [0.18,0.72]***	0.35 [0.18,0.69]***
Days on tracker [250–299]	0.25 [0.11,0.55]***	0.24 [0.11,0.53]***
Days on tracker [300–349]	0.25 [0.10,0.59]***	0.23 [0.10,0.56]***
Days on tracker [350–399]	0.23 [0.08,0.66]***	0.22 [0.08,0.62]***
Locality 7	1.00	1.00
Locality 6	2.31 [0.26,20.18]	1.44 [0.16,13.14]
Locality 5	0.90 [0.12,6.66]	1.04 [0.13,8.05]
Locality 4	0.52 [0.04,7.15]	0.47 [0.03,6.61]
Locality 3	2.55 [0.22,28.97]	2.71 [0.23,31.72]
Locality 2	9.67 [1.27,73.62]**	6.68 [0.85,52.59]*
Locality 1	25.49 [3.24,200.65]***	29.00 [3.67,229.20]***
Not-for-profit homes (vs for-profit homes)	0.65 [0.15,2.80]	0.69 [0.16,2.90]
Residential homes (vs nursing homes)	0.63 [0.30,1.35]	0.75 [0.32,1.73]
Small homes [1–23 occupied beds]	1.00	1.00
Medium homes [24–40 occupied beds]	1.19 [0.83,1.70]	1.16 [0.81,1.66]
Large homes [41+ occupied beds]	1.01 [0.64,1.59]	0.98 [0.62,1.54]
Chains owned homes (vs independent homes)	0.34 [0.17,0.69]***	0.30 [0.14,0.63]***
Homes with residents with dementia (yes/no flag)	0.67 [0.29,1.55]	0.51 [0.23,1.17]
Homes with learning disability and/or autism (yes/no flag)	0.36 [0.11,1.20]*	0.37 [0.11,1.23]
Homes with residents with physical disabilities (yes/no flag)	1.24 [0.63,2.42]	1.21 [0.63,2.31]
CQC rated inadequate	1.00	1.00
Requires improvement	0.49 [0.10,2.47]	0.45 [0.09,2.21]
Good	0.55 [0.13,2.31]	0.44 [0.10,2.01]
Outstanding	0.32 [0.06,1.78]	0.23 [0.04,1.39]
Homes COVID-19 +ve residents (%)		0.99 [0.99,1.00]*
Number of workers available for work		0.99 [0.98,1.00]
Number of MSOA level COVID-19 cases		1.00 [0.99,1.00]
Number of staff self-isolating		0.98 [0.94,1.02]
PPE [Less than a week]		1.00
PPE [1 to 4 weeks]		0.59 [0.32,1.11]
PPE [More than 1 month]		0.60 [0.33,1.11]
Accepting new admissions [No issue]		1.00
Accepting new admissions [Limited capacity]		1.03 [0.85,1.25]
Accepting new admissions [Emergencies only]		1.06 [0.68,1.67]
Accepting new admissions [Not possible]		1.12 [0.96,1.30]
Home FE		
Week FE	✓	✓
Interaction terms (Days on tracker x Locality)	✓	✓
Homes	130	130
N	20456	20456

Notes: *** p<0.01, ** p<0.05, * p<0.1. Cluster robust 95% confidence intervals are in the brackets. The dependent variable is tracker use [=1 if at least one resident is assessed on a given day in a care home, 0 otherwise]. The coefficient of one locality is not reported due to very low uptake. 7 homes are excluded due to missing CQC data. The estimations also include a categorical variable on home local area index of multiple deprivation; the coefficients are mostly insignificant and omitted to save space. The results were obtained from logistic regressions. To prevent omitted variables bias Column 2 includes additional variables and their means (means not reported). Almost all of the interactions (Days on tracker x Locality) were insignificant and not reported.

Appendix S3

Estimation method selection for estimating the odds of COVID-19 outbreaks

COVID-19 Outbreaks For the COVID-19 outbreaks the binary variable was regressed on a number of variables along with weeks and local authority dummies for all the care homes in the sample (manuscript: Columns 1 and 2 of Table 3). Since we were interested in retrieving the effects of homes legal/residential/ownership statuses, correlation random effect was our preferred estimation model for reasons discussed in Appendix 2.

Missing data In the COVID-19 outbreaks analysis, 50 out of 547 care homes were dropped. Among those, 31 could not be linked to CQC register, 19 care homes were dropped due to missing data on either local area index of multiple deprivations, CQC rating or complete missing data of the dependent variable. On the rest of variables used in the analysis, the combined missing data were less than 1%. Care homes quality ratings and local areas socio-economic status is shown to have significant associate of COVID-19 outbreaks in care homes.[4] Thus, dropping the rating and local deprivation index variables was not possible as that would have biased the coefficients of the other home characteristics.

Appendix S4

Estimation methods and sensitivity analysis tables for the tracker impact on COVID-19 spread

COVID-19 impacts of tracker For the tracker impact evaluation, we compared pre and post-tracker use periods changes in residents' COVID-19 outcomes for those care homes that opted the tracker during 2020 with care homes in the GM that did not opt for the tracker. Such Difference-in-Difference (DID) analysis assumes that, conditional on the other covariates in the model, over time changes in COVID-19

[4]Dykgraaf, S. H., Matenge, S., Desborough, J., Sturgiss, E., Dut, G., Roberts, L., ... & Kidd, M. (2021). Protecting nursing homes and long-term care facilities from COVID-19: a rapid review of international evidence. Journal of the American Medical Directors Association, 22(10), 1969-1988.

would have been the same in the intervention group as in the control group in the absence of the intervention (parallel trends assumption). In practice, one can only test pre-intervention parallel trends to assess the plausibility of this assumption. The DID analyses were conducted with 1) standard Poisson regression, 2) event design Poisson regression, and 3) weighting-based non/semi-parametric DID estimations.

In the standard fixed effects Poisson regression (Columns 3-5 of Table 3), the intervention is defined in terms of a single indicator taking a value of 1 for the post-intervention period for the homes in the intervention group only, zero elsewhere. However, recent studies have shown that regression-based DID analysis with such an indicator may lead to biased result, even if the parallel trends assumption holds, when there is variation in the intervention timings as is our case.[5]

In an event design, the event variable measures time relative to the intervention/event date (tracker uptake date in our case). Event indicators are then created from the event variable. Event design solves the bias that can arise from heterogenous treatment effects by avoiding the aggregation of these effects into a single treatment/effects coefficient, it also avoids the use of pre-intervention observations of care homes in the intervention group as counterfactuals. Event design gives a clear picture of whether the pre-intervention parallel trend holds, and whether the intervention has any dynamic effects on the outcomes.[6] Except Columns 1-2 of Table A6, all the DID estimates in the Appendix 4 come from event design.

As a further robustness check, we also implemented the non/semi-parametric[7] estimation approach of Callaway and Sant'Anna.[8] This approach allows for treatment heterogeneity across both care homes and over time. With daily data having many homes, it involved estimations of thousands of two-period x two-groups (2x2) treatment effects. To get around this, we aggregated the data to weekly-levels. The 2x2

[5]Goodman-Bacon, A. (2021). Difference-in-differences with variation in treatment timing. Journal of Econometrics, 225(2), 254-277.

[6]For the difference between the two methods compare Equations 1 and 6 (also a detailed example on page 5) of Clarke, D., and Schythe, K. (2020). Implementing the panel event study. IZA Discussion Paper No. 13524, Institute of Labor Economics (IZA). https://ssrn.com/abstract=3660271.

[7]A weak point of any regression based DID analysis is the parametric assumptions, i.e. the fixed effects and the error term is additively separable of the other confounders. Non-parametric estimations do not impose any functional form restrictions.

[8]Callaway, B., & Sant'Anna, P. H. (2021). Difference-in-differences with multiple time periods. Journal of Econometrics, 225(2), 200-230.

Table A3: DID estimates of COVID-19 cases per 100 beds in the pre and post tracker use periods

	Event design Poisson	Event design Poisson	Event design Poisson	Event design Poisson
Prior 34th day	1.24	1.50	3.22**	1.33
	[0.54,2.84]	[0.63,3.56]	[1.15,9.01]	[0.60,2.95]
28 days to event	1.16	1.07	1.10	1.23
	[0.34,3.93]	[0.31,3.74]	[0.22,5.58]	[0.38,3.98]
21 days to event	1.03	1.05	1.21	1.09
	[0.34,3.07]	[0.38,2.90]	[0.54,2.71]	[0.39,3.03]
14 days to event	1.53	1.49	1.30	1.57
	[0.65,3.58]	[0.68,3.27]	[0.58,2.92]	[0.68,3.60]
7 days to event	1.44	1.39	1.59	1.39
	[0.88,2.37]	[0.86,2.25]	[0.79,3.19]	[0.94,2.06]
1 day to event	1.18	1.35	1.77	1.36
	[0.76,1.83]	[0.82,2.20]	[0.82,3.83]	[0.86,2.17]
1 day after event	0.80	0.83	1.07***	0.84
	[0.53,1.21]	[0.51,1.37]	[1.03,1.11]	[0.51,1.38]
7 days after event	0.68	0.70	0.82	0.70
	[0.37,1.26]	[0.35,1.39]	[0.47,1.44]	[0.32,1.53]
14 days after event	0.40**	0.38**	0.51	0.45*
	[0.19,0.85]	[0.17,0.86]	[0.19,1.42]	[0.20,1.02]
21 days after event	0.83	0.73	0.55	0.90
	[0.24,2.87]	[0.19,2.75]	[0.13,2.28]	[0.24,3.32]
28 days after event	1.10	0.78	1.26	0.85
	[0.27,4.48]	[0.19,3.13]	[0.37,4.32]	[0.22,3.31]
Beyond 34th day	1.08	1.05	1.67	1.03
	[0.43,2.75]	[0.39,2.81]	[0.56,5.00]	[0.44,2.41]
Number of workers available lag 7		0.98***	0.98***	0.99
		[0.97,0.99]	[0.97,0.99]	[0.98,1.00]
Workers available sqr. lag 7		1.00	1.00	1.00
		[1.00,1.00]	[1.00,1.00]	[1.00,1.00]
Number of beds available lag 7		1.01*	1.02	1.00
		[1.00,1.03]	[1.00,1.04]	[0.99,1.02]
Number of occupied beds lag 7		0.99**	1.00	0.99**
		[0.97,1.00]	[0.98,1.02]	[0.97,1.00]
Accepting new admissions [No issue] lag 7		1	1	1
		[1.00,1.00]	[1.00,1.00]	[1.00,1.00]
Accepting new admissions [Limited capacity] lag 7		1.26	1.77***	1.21
		[0.86,1.85]	[1.20,2.62]	[0.84,1.76]
Accepting new admissions [Emergencies only] lag 7		0.92	1.65	0.95
		[0.33,2.54]	[0.67,4.03]	[0.48,1.87]
Accepting new admissions [Not possible] lag 7		1.73***	3.23***	1.56**
		[1.20,2.51]	[1.87,5.59]	[1.11,2.21]
Worker RAG [Green] lag 7		1	1	1
		[1.00,1.00]	[1.00,1.00]	[1.00,1.00]
Worker RAG [Amber] lag 7		2.13***	1.66***	1.19
		[1.70,2.68]	[1.32,2.09]	[0.93,1.52]
Worker RAG [Red] lag 7		4.28***	3.80***	1.42*
		[3.07,5.97]	[2.40,6.00]	[1.00,2.01]
Number of MSOA level COVID-19 cases lag 7		1.06***	1.08***	1.05***
		[1.04,1.08]	[1.06,1.11]	[1.04,1.07]
MSOA level COVID-19 cases sqr. lag 7		1.00***	1.00***	1.00***
		[1.00,1.00]	[1.00,1.00]	[1.00,1.00]
Number of staff self-isolating lag 7				1.29***
				[1.22,1.36]
Staff self-isolating sqr. lag 7				0.99***
				[0.99,1.00]
PPE [Less than a week				1] lag 7
				[1.00,1.00]
PPE [1 to 4 weeks] lag 7				0.74**
				[0.55,0.99]
PPE [More than 1 month] lag 7				0.86
				[0.47,1.57]
N	98890	95033	41245	95026
Homes	410	406	174	406
Pseudo R-squared	0.314	0.381	0.394	0.434

Notes: *** p<0.01, ** p<0.05, * p<0.1. Cluster robust 95% confidence intervals are in the brackets. The coefficients are incidence rate ratios. All the control variables are included with lag 7 keeping in view the incubation period of COVID-19. Column 3 estimations were run on a matched sub-sample (matching was done on all the characteristics reported in the CQC data given in manuscript Table 1 with nearest 5 neighbours for each treated home). The estimations included 34 pre and 34 post uptake event indicators, but to save space reports only the ones with seven days gap.

intervention effects were then aggregated using time-to-intervention as weights. Column 1 of Table A4 reports results for crude/unadjusted average treatment effects from this approach, whereas Columns 2 and 3 results are conditional average treatment effects (controlling for pre-intervention covariates).

Missing data 13 care homes were in the database but had no data on COVID-19 or occupied beds during 2020. The DID estimations included 534 care homes with 127,589 observations (homes x days). Over the 2020 period, 124 care homes had no positive/symptomatic residents, thus they were excluded from the DID estimations with Poisson regression (as the outcomes of such homes are predicted by the home indicator alone in probability-based regressions). Nevertheless, these homes were included in the non/semi-parametric estimations in Table A4.

Table A4: DID estimates of COVID-19 cases per 100 beds in the pre and post tracker use periods (weekly data)

	Non-parametric event design	Semi-parametric event design	Semi-parametric event design
15 weeks to event	-0.103	0.0240	-0.0521
	[-0.676,0.471]	[-0.730,0.778]	[-1.617,1.513]
12 weeks to event	0.524	0.739	1.190
	[-0.517,1.564]	[-0.603,2.080]	[-1.082,3.462]
9 weeks to event	-0.0227	-0.0569	0.0475
	[-0.411,0.366]	[-0.756,0.643]	[-2.950,3.045]
6 weeks to event	1.535	2.283**	2.542
	[-0.312,3.382]	[0.195,4.371]	[-2.465,7.549]
5 weeks to event	1.207	1.521	2.274
	[-0.804,3.217]	[-0.854,3.896]	[-3.635,8.183]
4 weeks to event	-0.260	0.259	0.289
	[-1.479,0.960]	[-1.248,1.765]	[-2.769,3.346]
3 weeks to event	-0.216	-0.785	-2.189
	[-1.782,1.351]	[-3.019,1.448]	[-6.388,2.011]
2 weeks to event	1.051*	1.711	0.136
	[-0.124,2.226]	[-1.277,4.698]	[-3.068,3.339]
1 week to event	-0.680	-0.456	-1.364
	[-2.960,1.599]	[-3.337,2.425]	[-5.832,3.104]
Event week	-0.500	-0.882	0.00561
	[-1.370,0.370]	[-2.243,0.480]	[-4.095,4.106]
1 week post event	-0.792	-2.098	-1.582
	[-2.073,0.489]	[-4.786,0.590]	[-4.829,1.664]
2 weeks post event	-0.931	-3.918**	-0.976
	[-2.106,0.244]	[-7.335,-0.500]	[-3.986,2.033]
3 weeks post event	-0.582	-3.717	-0.724
	[-2.652,1.488]	[-8.300,0.867]	[-3.929,2.481]
4 weeks post event	-0.656	-2.401	-1.011
	[-3.362,2.049]	[-7.971,3.169]	[-5.549,3.527]
5 weeks post event	0.240	0.455	-1.425
	[-2.766,3.246]	[-7.705,8.614]	[-11.207,8.357]
6 weeks post event	-0.366	-0.160	-0.531
	[-3.027,2.294]	[-7.040,6.721]	[-9.056,7.993]
7 weeks post event	-1.609	-4.147	-1.558
	[-3.858,0.639]	[-9.334,1.041]	[-9.120,6.004]
8 weeks post event	-1.903	-5.240	-3.328
	[-4.409,0.603]	[-11.494,1.013]	[-10.657,4.001]
9 weeks post event	-2.424**	-6.088*	-4.490
	[-4.738,-0.110]	[-12.281,0.106]	[-10.794,1.813]
12 weeks post event	-2.169*	-4.511	-1.335
	[-4.608,0.269]	[-10.026,1.003]	[-7.050,4.380]
15 weeks post event	0.588	-1.135	-1.567
	[-3.724,4.899]	[-10.997,8.727]	[-14.155,11.021]
18 weeks post event	-1.905	-8.292	1.776
	[-4.242,0.433]	[-20.604,4.019]	[-18.716,22.267]
21 weeks post event	-0.843	-9.228	30.79
	[-4.518,2.832]	[-25.396,6.939]	[-23.509,85.081]
24 weeks post event	3.255	-11.32	49.93
	[-7.426,13.936]	[-28.814,6.181]	[-42.662,142.515]
Observations	17,909	16,957	15,514

Notes: *** p<0.01, ** p<0.05 * p<0.1. Bootstrap (999 repetitions) 95% confidence intervals are in the brackets. These non/semi-parametric results come from Callaway and Sant'Anna approach on weekly data. Column 2 adjusts for the pre tracker use values of all confounders in Table A3. Column 3 also adjusts for the pre tracker use values of all the variables in manuscript Table 1 including home CQC rating and home area index of multiple deprivation. Adjustment is done through propensity scores re-weighting. The estimations included all pre and post uptake event weeks, but to save space all are not reported.

Table A5: DID estimates of COVID-19 cases per 100 beds (parsimonious to general specification)

	Poisson	Poisson	Poisson	Poisson	Poisson	Poisson
Prior 34th day	1.27	1.23	1.25	1.32	1.50	1.33
	[0.49,3.25]	[0.50,3.04]	[0.51,3.09]	[0.55,3.17]	[0.63,3.56]	[0.60,2.95]
28 days to event	1.03	1.00	0.99	0.97	1.07	1.23
	[0.27,3.92]	[0.27,3.72]	[0.27,3.70]	[0.28,3.40]	[0.31,3.74]	[0.38,3.98]
21 days to event	0.91	0.87	0.87	0.92	1.05	1.09
	[0.28,2.90]	[0.28,2.71]	[0.28,2.73]	[0.34,2.47]	[0.38,2.90]	[0.39,3.03]
14 days to event	1.28	1.21	1.25	1.23	1.49	1.57
	[0.52,3.14]	[0.50,2.93]	[0.51,3.02]	[0.58,2.59]	[0.68,3.27]	[0.68,3.60]
7 days to event	1.29	1.27	1.31	1.31	1.39	1.39
	[0.80,2.08]	[0.82,1.99]	[0.83,2.04]	[0.82,2.10]	[0.86,2.25]	[0.94,2.06]
1 day to event	1.30	1.28	1.31	1.36	1.35	1.36
	[0.82,2.08]	[0.81,2.02]	[0.83,2.06]	[0.84,2.21]	[0.82,2.20]	[0.86,2.17]
1 day after event	0.85	0.85	0.85	0.84	0.83	0.84
	[0.51,1.42]	[0.51,1.41]	[0.51,1.41]	[0.51,1.38]	[0.51,1.37]	[0.51,1.38]
7 days after event	0.62	0.64	0.60	0.61	0.70	0.70
	[0.29,1.35]	[0.31,1.35]	[0.28,1.32]	[0.29,1.29]	[0.35,1.39]	[0.32,1.53]
14 days after event	0.37**	0.36**	0.36**	0.34**	0.38**	0.45*
	[0.15,0.92]	[0.15,0.89]	[0.14,0.88]	[0.14,0.80]	[0.17,0.86]	[0.20,1.02]
21 days after event	0.67	0.69	0.67	0.67	0.73	0.90
	[0.17,2.68]	[0.18,2.67]	[0.17,2.58]	[0.18,2.55]	[0.19,2.75]	[0.24,3.32]
28 days after event	0.85	0.81	0.82	0.72	0.78	0.85
	[0.18,4.11]	[0.17,3.77]	[0.18,3.78]	[0.16,3.17]	[0.19,3.13]	[0.22,3.31]
Beyond 34th day	0.96	0.89	0.89	0.87	1.05	1.03
	[0.32,2.91]	[0.31,2.59]	[0.30,2.61]	[0.32,2.38]	[0.39,2.81]	[0.44,2.41]
Number of MSOA level COVID-19 cases lag 7	1.07***	1.06***	1.06***	1.06***	1.06***	1.05***
	[1.04,1.09]	[1.04,1.09]	[1.04,1.09]	[1.04,1.09]	[1.04,1.08]	[1.04,1.07]
MSOA level COVID-19 cases sqr. lag 7	1.00***	1.00***	1.00***	1.00***	1.00***	1.00***
	[1.00,1.00]	[1.00,1.00]	[1.00,1.00]	[1.00,1.00]	[1.00,1.00]	[1.00,1.00]
Number of workers available lag 7		0.98***	0.98***	0.98***	0.98***	0.99
		[0.97,0.99]	[0.97,0.99]	[0.97,0.98]	[0.97,0.99]	[0.98,1.00]
Workers available sqr. lag 7		1.00	1.00	1.00*	1.00	1.00
		[1.00,1.00]	[1.00,1.00]	[1.00,1.00]	[1.00,1.00]	[1.00,1.00]
Number of beds available lag 7			1.01	1.01*	1.01*	1.00
			[0.99,1.03]	[1.00,1.03]	[1.00,1.03]	[0.99,1.02]
Number of occupied beds lag 7			0.98***	0.98**	0.99**	0.99**
			[0.97,0.99]	[0.97,1.00]	[0.97,1.00]	[0.97,1.00]
Accepting new admissions [No issue] lag 7				1	1	1
				[1.00,1.00]	[1.00,1.00]	[1.00,1.00]
Accepting new admissions [Limited capacity] lag 7				1.28	1.26	1.21
				[0.86,1.90]	[0.86,1.85]	[0.84,1.76]
Accepting new admissions [Emergencies only] lag 7				1.14	0.92	0.95
				[0.47,2.75]	[0.33,2.54]	[0.48,1.87]
Accepting new admissions [Not possible] lag 7				1.93***	1.73***	1.56**
				[1.33,2.82]	[1.20,2.51]	[1.11,2.21]
Worker RAG [Green] lag 7					1	1
					[1.00,1.00]	[1.00,1.00]
Worker RAG [Amber] lag 7					2.13***	1.19
					[1.70,2.68]	[0.93,1.52]
Worker RAG [Red] lag 7					4.28***	1.42*
					[3.07,5.97]	[1.00,2.01]
Number of staff self-isolating lag 7						1.29***
						[1.22,1.36]
Staff self-isolating sqr. lag 7						0.99***
						[0.99,1.00]
N	95836	95774	95767	95033	95033	95026
Pseudo R	0.342	0.353	0.356	0.363	0.381	0.434

Notes: *** p<0.01, ** p<0.05, * p<0.1. Cluster robust 95% confidence intervals are in the brackets. The coefficients are incidence rate ratios. All the control variables are included with lag 7 keeping in view the incubation period of COVID-19. The estimations included 34 pre and 34 post uptake event indicators, but to save space reports only the ones with seven days gap.

Table A6: DID estimates of COVID-19 cases per 100 beds (only 4 localities)

VARIABLES	Standard Linear	Standard Poisson	Event design Poisson	Event design Linear	Event design Poisson
Tracker uptake lag 7	0.42*	0.45***			
	[0.16,1.11]	[0.26,0.78]			
Prior 34th day			1.51	4.93	1.14
			[0.64,3.58]	[0.44,55.57]	[0.25,5.13]
28 days to event			1.92	102.1	1.56
			[0.58,6.32]	[0.03,355426.84]	[0.25,9.64]
21 days to event			1.42	41.9	1.23
			[0.46,4.41]	[0.05,33233.54]	[0.30,5.09]
14 days to event			2.68*	57.7	1.82
			[0.95,7.54]	[0.04,91452.60]	[0.31,10.59]
7 days to event			1.94*	2.67*	0.87
			[0.97,3.88]	[0.88,8.07]	[0.45,1.69]
1 day to event			1.03	1.91*	1.10
			[0.88,1.19]	[0.89,4.10]	[0.76,1.59]
1 day after event			0.77	1.16	1.17
			[0.42,1.43]	[0.90,1.50]	[0.91,1.49]
7 days after event			0.76	1.21	0.95
			[0.32,1.80]	[0.25,5.87]	[0.33,2.76]
14 days after event			0.32**	0.86	0.31
			[0.12,0.87]	[0.12,6.19]	[0.08,1.30]
21 days after event			0.54	1.14	0.38
			[0.17,1.67]	[0.12,10.97]	[0.04,3.44]
28 days after event			0.36	3.62	0.83
			[0.09,1.44]	[0.29,45.42]	[0.20,3.43]
Beyond 34th day			0.75	5.01	0.66
			[0.32,1.74]	[0.39,63.55]	[0.17,2.66]
Number of staff self-isolating lag 7	4.23***	1.48***	1.47***	4.72***	1.53***
	[2.77,6.47]	[1.34,1.62]	[1.34,1.61]	[2.19,10.17]	[1.30,1.80]
Staff self-isolating sqr. lag 7	0.96***	0.98***	0.98***	0.95***	0.98***
	[0.94,0.98]	[0.98,0.99]	[0.98,0.99]	[0.92,0.98]	[0.97,0.99]
Number of workers available lag 7	0.84***	0.95***	0.95***	0.85**	0.96**
	[0.76,0.93]	[0.91,0.98]	[0.92,0.98]	[0.73,1.00]	[0.93,0.99]
Workers available sqr. lag 7	1.00***	1.00	1.00	1.00*	1.00
	[1.00,1.00]	[1.00,1.00]	[1.00,1.00]	[1.00,1.00]	[1.00,1.00]
Number of beds available lag 7	0.98	0.99	1.00	1.02	1.00
	[0.94,1.03]	[0.98,1.01]	[0.98,1.01]	[0.95,1.10]	[0.96,1.04]
Number of occupied beds lag 7	0.94	0.98**	0.98*	0.98	0.99
	[0.88,1.01]	[0.97,1.00]	[0.97,1.00]	[0.91,1.06]	[0.96,1.02]
Accepting new admissions [No issue] lag 7	1	1	1	1	1
	[1.00,1.00]	[1.00,1.00]	[1.00,1.00]	[1.00,1.00]	[1.00,1.00]
Accepting new admissions [Limited capacity] lag 7	1.77	1.51	1.50	2.17	1.47
	[0.70,4.43]	[0.89,2.56]	[0.89,2.54]	[0.47,9.91]	[0.58,3.75]
Accepting new admissions [Emergencies only] lag 7	0.54	0.49	0.47	0.17	1
	[0.02,11.74]	[0.06,3.78]	[0.06,3.42]	[0.00,16.31]	[1.00,1.00]
Accepting new admissions [Not possible] lag 7	4.72**	2.14***	2.15***	9.43*	2.35*
	[1.34,16.63]	[1.30,3.52]	[1.34,3.44]	[0.65,136.12]	[0.99,5.60]
Worker RAG [Green] lag 7	1	1	1	1	1
	[1.00,1.00]	[1.00,1.00]	[1.00,1.00]	[1.00,1.00]	[1.00,1.00]
Worker RAG [Amber] lag 7	2.94	0.82	0.84	26.1	1.10
	[0.27,31.89]	[0.41,1.64]	[0.42,1.67]	[0.01,46385.49]	[0.38,3.20]
Worker RAG [Red] lag 7	1406.9*	1.99*	2.11*	133.1	1.62
	[0.28,7174878.61]	[0.92,4.32]	[0.98,4.55]	[0.00,41628061.14]	[0.32,8.32]
PPE [Less than a week] lag 7	1	1	1	1	1
	[1.00,1.00]	[1.00,1.00]	[1.00,1.00]	[1.00,1.00]	[1.00,1.00]
PPE [1 to 4 weeks] lag 7	0.28	0.71	0.70	0.41	0.67
	[0.05,1.70]	[0.37,1.36]	[0.36,1.35]	[0.02,8.96]	[0.18,2.50]
PPE [More than 1 month] lag 7	0.23	0.66	0.61	0.11	0.26
	[0.03,1.89]	[0.26,1.69]	[0.24,1.53]	[0.00,5.49]	[0.04,1.76]
Number of MSOA level COVID-19 cases lag 7	1.12***	1.05***	1.05***	1.17***	1.06***
	[1.04,1.20]	[1.02,1.08]	[1.02,1.07]	[1.08,1.27]	[1.03,1.10]
MSOA level COVID-19 cases sqr. lag 7	1.00**	1.00**	1.00**	1.00***	1.00***
	[1.00,1.00]	[1.00,1.00]	[1.00,1.00]	[1.00,1.00]	[1.00,1.00]
N	42378	35270	35270	18542	15702
R/Pseudo R-squared	0.122	0.414	0.416	0.163	0.447
Number of homes	183	151	151	79	66

Notes: *** p<0.01, ** p<0.05, * p<0.1. Cluster robust 95% confidence intervals are in the brackets. These results are for the 4 localities (1-4) that opted the tracker before mid-November 2020. Columns 4 and 5 estimations are run on a matched sub-sample (matching is done on all the characteristics reported in the CQC data given in manuscript Table 1 with nearest neighbours for each treated home). Rest of the details are as in Table A3.

Table A7: DID estimates of COVID-19 cases per 100 beds (4 localities only and includes intensity of use)

	Poisson	Poisson	Poisson
Assessed(%) lag 5			1.00
			[1.00,1.00]
Assessed(%) lag 6			1.00
			[1.00,1.00]
Assessed(%) lag 7			1.00
			[1.00,1.00]
Prior 21st day	1.34	1.53	1.35
	[0.61,2.93]	[0.18,12.60]	[0.61,2.98]
14 days to event	2.37*	2.13	2.37*
	[0.98,5.72]	[0.22,20.41]	[0.98,5.76]
7 days to event	1.65**	0.91	1.63*
	[1.01,2.69]	[0.27,3.09]	[0.99,2.68]
1 day to event	1.05	1.61	1.06
	[0.89,1.25]	[0.65,3.95]	[0.89,1.25]
1 day after event	0.78	1.11	0.78
	[0.42,1.45]	[0.94,1.31]	[0.42,1.44]
7 days after event	0.68	1.22	0.69
	[0.30,1.55]	[0.23,6.58]	[0.27,1.72]
14 days after event	0.27***	0.68	0.27**
	[0.11,0.70]	[0.32,1.43]	[0.10,0.77]
Beyond 21st day	0.63	0.86	0.64
	[0.29,1.37]	[0.10,7.72]	[0.28,1.46]
Staff self-isolating lag 7	1.46***	1.52***	1.46***
	[1.33,1.59]	[1.29,1.79]	[1.33,1.60]
Staff self-isolating sqr. lag 7	0.98***	0.98***	0.98***
	[0.98,0.99]	[0.97,0.99]	[0.98,0.99]
Number of workers available lag 7	0.95***	1.02	0.95***
	[0.92,0.98]	[0.95,1.09]	[0.92,0.99]
Workers available sqr. lag 7	1.00	1.00	1.00
	[1.00,1.00]	[1.00,1.00]	[1.00,1.00]
Number of beds available lag 7	0.99	1.00	0.99
	[0.97,1.01]	[0.98,1.03]	[0.97,1.01]
Number of occupied beds lag 7	0.98**	0.97	0.98**
	[0.97,1.00]	[0.94,1.01]	[0.97,1.00]
Accepting new admissions lag 7	1.26***	1.34**	1.26***
	[1.07,1.48]	[1.03,1.74]	[1.07,1.48]
Worker RAG lag 7	1.19	2.12*	1.19
	[0.75,1.90]	[0.88,5.14]	[0.75,1.89]
PPE lag 7	0.85	0.81	0.85
	[0.70,1.04]	[0.59,1.12]	[0.70,1.04]
Number of MSOA level COVID-19 cases lag 7	1.05***	1.06***	1.05***
	[1.02,1.08]	[1.02,1.10]	[1.02,1.08]
MSOA level COVID-19 cases sqr. lag 7	1.00**	1.00**	1.00**
	[1.00,1.00]	[1.00,1.00]	[1.00,1.00]
N	35270	13030	35259
Pseudo R	0.415	0.427	0.415

Notes: *** $p<0.01$, ** $p<0.05$, * $p<0.1$. Cluster robust 95% confidence intervals are in the brackets. The coefficients are incidence rate ratios. Column 3 includes the intensity of use (daily percentage of residents assessed) besides the uptake dummies. Column 2 estimation are run on a matched sub-sample (matching is done on all the characteristics reported in the CQC data given in Table 1 with nearest neighbours for each treated unit).
